# Enhanced Ocular Bioavailability and Prolonged Duration via Hydrophilic Surface Nanocomposite Vesicles for Topical Drug Administration

**DOI:** 10.3390/pharmaceutics16121496

**Published:** 2024-11-21

**Authors:** Sa Huang, Yuan Xu, Yingyao Luo, Zhijiong Wang, Fan Li, Zhenmiao Qin, Junfeng Ban

**Affiliations:** 1 Guangdong Provincial Key Laboratory of Advanced Drug Delivery, Guangdong Provincial Engineering Center of Topical Precise Drug Delivery System, Guangdong Pharmaceutical University, No. 280 University Town Outer Ring East Road, Guangzhou 510006, China; 2 College of Pharmacy, Guilin Medical University, No. 1 Zhiyuan Road, Guilin 541104, China; 3 Guangdong Laboratory Animals Monitoring Institute, No. 11 Fengxin Road, Guangzhou 510663, China; 4 The Innovation Team for Integrating Pharmacy with Entrepreneurship, Guangdong Pharmaceutical University, No. 280 University Town Outer Ring East Road, Guangzhou 510006, China; 5 Key Laboratory of Tropical Translational Medicine of Ministry of Education, Hainan Key Laboratory for Research and Development of Tropical Herbs, School of Pharmacy, Hainan Medical University, No. 3 Xueyuan Road, Haikou 571199, China

**Keywords:** ocular drug delivery, nanoparticles, cell-penetrating peptides, PLGA, pharmacokinetic

## Abstract

Background: Internal ocular diseases, such as macular edema, uveitis, and diabetic macular edema require precise delivery of therapeutic agents to specific regions within the eye. However, the eye’s complex anatomical structure and physiological barriers present significant challenges to drug penetration and distribution. Traditional eye drops suffer from low bioavailability primarily due to rapid clearance mechanisms. Methods: The novel ocular drug delivery system developed in this study utilizes poly(lactic-co-glycolic acid) (PLGA) nanoparticles modified with cell-penetrating peptides (CPPs). In vitro drug release studies were conducted to evaluate the sustained-release properties of the nanoparticles. Ex vivo experiments using MDCK cells assessed corneal permeability and uptake efficiency. Additionally, in vivo studies were performed in rabbit eyes to determine the nanoparticles’ resistance to elimination by tears and their retention time in the aqueous humor. Results: In vitro drug release studies demonstrated superior sustained-release properties of the nanoparticles. Ex vivo experiments revealed enhanced corneal permeability and increased uptake efficiency by MDCK cells. In vivo studies in rabbit eyes confirmed the nanoparticles’ resistance to elimination by lacrimal fluid and their ability to extend retention time in the aqueous humor. CPP modification significantly improved ocular retention, corneal penetration, and cellular endocytosis efficiency. Conclusions: The CPP-modified PLGA nanoparticles provide an effective and innovative solution for ocular drug delivery, offering improved bioavailability, prolonged retention, and enhanced drug penetration, thereby overcoming the challenges of traditional intraocular drug administration methods.

## 1. Introduction

Treating Inflammatory fundus diseases such as macular edema, uveitis, and diabetic macular edema requires medication delivery to the affected eye parts [1,2]. The unique anatomical structure of the eyeball and physiological barriers, such as the cornea, blood–aqueous barrier, and blood–retinal barrier, limits drug penetration and distribution inside the eye [3]. The conventional ocular drug delivery modality, such as intraocular injection of triamcinolone acetonide (TA) is commonly used to treat these conditions [4,5,6]. The high risk associated with intravitreal injections has led to a preference for non-invasive formulations, such as ocular instillation. However, the transient residence time of the medication within the ocular surface is associated with the lacrimal fluid’s rinsing action and the subsequent nasolacrimal pathway drainage. This results in suboptimal bioavailability, often below 5% [7]. Non-invasive nano-drug delivery systems have generated substantial interest in traversing ocular barriers. These advanced formulations provide notable advantages in augmenting drug bioavailability, minimizing adverse effects, and improving drug stability and targeting capabilities. For instance, nanoparticles, nanoliposomes, and nanomicelles can improve the ocular surface penetration of pharmaceuticals, prolong drug retention times, elevate drug solubility, mitigate drug toxicity, extend drug release duration, and facilitate targeted delivery to ocular tissues [8]. In preliminary studies, a nano-drug delivery system was developed depending on PLGA as a carrier, and its ability to cross the ocular barriers was evaluated. The results depicted that the prepared nanoparticles could enhance the transmembrane permeability of drugs such as triamcinolone acetonide and dexamethasone while elevating their aqueous humor concentration. The mechanism could be associated with the carrier forming a drug reservoir, which slows down and sustains medication release [9,10]. The cell membrane is a formidable biological barrier hindering the drug molecule entry into cells. Barrier overcoming ensures the safe and effective delivery of drugs to their target sites while facilitating cellular entry. This is a considerable challenge for contemporary PLGA-based nano-drug delivery systems [11]. CPPs represent a distinct class of short peptides, which can traverse cell membranes. These peptides comprise fewer than 30 amino acid residues [12]. Recently, substantial advancements have been achieved in CPP research within the drug delivery domain. These advancements are particularly notable in enhancing cellular internalization and bioavailability while targeting the specificity of therapeutic agents [13,14]. Researchers are investigating innovative strategies to integrate CPPs with nanocarriers while examining their potential to overcome drug resistance and improve therapeutic efficacy. Steinbach et al. [15] used CPPs as surface modifiers for nanoparticles, leading to a 69-fold increase in nanoparticle internalization. Moreover, CPPs can directly bind to drug molecules and form CPP–drug complexes, thereby improving the cellular penetration of these drugs. Kamei et al. [14] aggregated insulin with CPPs, significantly enhancing insulin absorption within the intestine. Furthermore, CPPs are used for targeted drug delivery. For instance, Song et al. [16] conjugated doxorubicin with CPPs to selectively target breast cancer cells. CPPs can modify drug carriers like nanoliposomes, polymeric micelles, and inorganic nanoparticles, improving their cellular penetration and drug release efficiency. The current study determines the transmembrane capabilities and delivery efficiency of PLGA nanoparticles modified using CPPs for ocular barriers while optimizing non-invasive ocular drug delivery systems.

## 2. Materials and Methods

### 2.1. Materials

Triamcinolone acetonide active pharmaceutical ingredient (TA, 99.9%) was procured from Tianjin Jinhui Pharmaceutical Co., Ltd. (Tianjin, China). Triamcinolone acetonide reference substance (TA, 98.8%) was provided by China National Institutes for Food and Drug Control (NIFDC); Polyvinyl alcohol (PVA) from Kuraray International Trading Co., Ltd. (Shanghai, China); Poly(lactic-co-glycolic acid) copolymer (PLGA, 50:50, Mw 10,000 Da) from JFK Biotechnology Co., Ltd. (Jinan, China); 2-Hydroxypropyl-β-cyclodextrin (2-HP-β-CD, substitution degree 4.96) from Qianhui Biotechnology Co., Ltd. (Zibo, China); and CPP RK-16 from GL Biochem (Shanghai) Co., Ltd. (Shanghai, China); Ulatan (China National Pharmaceutical Group Corporation Chemical Reagent Company Limited, Shanghai, China); Tobramycin eye drops (Santen Pharmaceutical Co., Ltd., Suzhou, China); Filipin, Nystatin, Brefeldin A, and Monensin were bought from Dalian Meilun Biotech Co., Ltd. (Dalian, China); EIPA (Ethylisopropyl amiloride) and Chlorpromazine hydrochloride were acquired from MedChemExpress, Monmouth Junction, NJ, USA. All the other chemicals and organic solvents were analytical reagents (ARs).

### 2.2. Preparation of Nanocomposite Vesicles of Triamcinolone Acetonide

The solvent evaporation method helped prepare the formulation [17,18]. The prescriptions for each preparation are given in Table 1. Triamcinolone acetonide and PLGA (1:10, *m*/*m*) were dissolved in a mixture of acetone and dichloromethane (3:2, *v*/*v*) as the organic phase. At the same time, 1.5% 2-HP-β-CD and a 2% PVA aqueous solution became the aqueous phase. Ultrasonic emulsification (using a 6 mm probe at 150 kHz) was performed under ice bath conditions (0–4 °C) while slowly adding the organic phase into the aqueous phase over 0.5 h. The organic solvents were evaporated at room temperature. This was followed by adding double-distilled water to adjust the pH between 7.0 and 7.5, forming PLGA nanoparticle eye drops. A comparative study was performed to elucidate the effects of CPP integration on the permeability of formulations in vitro and in vivo. A series of PLGA nanoparticles devoid of CPP were synthesized using a methodology analogous to standard PLGA nanoparticles. The sole variation was the absence or presence of CPP within the aqueous phase. Subsequently, as mentioned during synthesis, the fluorescent probe synthesis was initiated, predicated on the nanoparticle, and incorporated Coumarin-6 to delineate the cellular uptake kinetics.

### 2.3. Characterization of Nanocomposite Vesicles of Triamcinolone Acetonide

#### 2.3.1. Size and Zeta Potential for Nanoparticles

The morphological attributes of the synthesized nanoparticles were scrutinized using a high-resolution transmission electron microscope (JEM-2100F, Carl Zeiss, Jena, Germany). A minute volume of nanoparticle suspension was applied onto a carbon-coated copper grid and stained with a 2% phosphotungstic acid solution for 10 min. Subsequently, the grid was air-dried at ambient temperature for more than 12 h to ensure complete solvent evaporation. Then, the specimens were examined under an electron microscope.The dimensions, polydispersity index (PDI), and zeta potential of the nanoparticles were ascertained with a nanoparticle size and zeta potential analyzer (Delsa Nano C, Beckman Coulter, Brea, CA, USA). The current analysis was performed to discern variations in particle size and surface charge post-CPP modification. The sample was properly diluted using deionized water before the assay to obtain optimal measurement conditions. The temperature inside the cuvette was meticulously maintained at 25 ± 1 °C throughout the assay to ensure experimental consistency.

#### 2.3.2. Physical Properties of Eye Drops

The surface tension of ocular formulations is a critical determinant of adhesion and spreading on the cornea, which is pivotal for corneal retention. Optimal surface tension can improve the residence time of corneal eye drops. We quantified the surface tension of the formulations to determine the propensity of eye drops while inducing blinking and tearing post-administration. The surface tension of various formulations was assessed at room temperature using the hanging drop method with an optical contact angle meter (SDC-200, Dongguan Shengding Precision Instruments Co., Ltd., Dongguan, China). A pH meter (FE28, Mettler Toledo, Zurich, Switzerland) was employed to measure the pH. At the same time, a freezing point osmometer (FM-9X, Shanghai Beijing Industrial Co., Ltd., Shanghai, China) helped ascertain the osmotic pressure of the CPP nanoparticle solution before and after modification.

#### 2.3.3. Loading Capacity and Entrapment Efficiency

The loading capacity dosage and encapsulation efficiency are pivotal metrics to determine the efficacy of nanoscale drug delivery systems. Triamcinolone acetonide quantification inside the nanoparticles was performed using high-performance liquid chromatography (Agilent 1260 HPLC apparatus, Santa Clara, CA, USA). The chromatographic separation was achieved using a Kromasil 100-5 C18 column (E25643, 5 μm, 250 mm × 4.6 mm). The mobile phase comprised a binary mixture of water and acetonitrile in a ratio of 58:42, and the flow rate was maintained at 1.0 $\mathrm{mL}\min^{-1}$. The detection was performed at a 240 nm wavelength with an injection volume standardized to 20 μL. Preliminary nanoparticle separation from the particulate form of the undissolved drug was accomplished through low-speed centrifugation. This induced the sedimentation of the undissolved drug at the bottom of the vessel, with the supernatant enriched with nanoparticles and a minor dissolved drug concentration. Then, the supernatant was conveyed into an ultrafiltration device to effectuate the discrete separation of the dissolved drug from the nanoparticles. The encapsulation efficiency (*EE*) and drug loading (*DL*) of the nanoparticle system were measured with the formula follows.

(1) $$EE\left(\%\right)=\frac{w_{1}-w_{2}}{w_{o}}\times 100\%$$

(2) $$DL\left(\%\right)=\frac{\left(w_{1}-w_{2}\right)\times V}{M}\times 100\%$$

In the given equation, $W_{o}$ signifies the pristine drug concentration, $w_{1}$ signifies the drug concentration in the supernatant phase post-low-speed centrifugation, $w_{2}$ indicates the drug concentration in the ultrafiltrate, *V* denotes the cumulative nanoformulation volume, and *M* represents the aggregate quantity of PLGA encapsulated in the nanoformulation.

### 2.4. In Vitro Drug Release and Evaluation of Corneal Permeability

We conducted in vitro release assays and corneal permeation studies to prognosticate the ocular bioavailability and the in vivo nanoparticle distribution while characterizing the drug release kinetics.

#### 2.4.1. In Vitro Drug Release

The dialysis bag technique helped assess the in vitro release kinetics of nanoparticles with and without CPP modification [19]. Artificial aqueous humor was formulated to emulate the intraocular milieu with higher fidelity, comprising 10.0 mmol L^−1^ HEPES, 136.2 mmol L^−1^ sodium chloride, 5.3 mmol L^−1^ potassium chloride, 1.0 mmol L^−1^ dipotassium phosphate, 1.7 mmol L^−1^ calcium chloride, and 5.5 mmol L^−1^ glucose in a buffer solution. The pH was adjusted to 7.4 at 37 °C, using 1.0 mmol L^−1^ sodium chloride solution. To simulate the drug release conditions in vivo, the experimental setup was conducted at a temperature of 34 ± 0.5 °C, highlighting the physiological conditions of the aqueous humor [20,21]. A 2 mL nanoformulation aliquot, post- and pre-CPP modification, was encapsulated inside a dialysis membrane. It was secured at both ends and immersed in a 50 mL centrifuge tube containing 25 mL of the artificial aqueous humor. The incubation conditions were maintained at 100 rpm and a controlled 34 ± 0.5 °C temperature with constant shaking. At predefined time intervals of 1, 2, 4, 6, 8, and 12 h, a 2 mL aliquot of the release medium was extracted and replenished using an equal volume of the pre-equilibrated blank medium. The samples were passed through a 0.22 μm filter. Moreover, the TA concentration was quantified using the analytical procedure delineated in Section 2.3.3. The cumulative percent release of triamcinolone acetonide was computed using the following formula:

(3) $$Q=\frac{C_{n}\times 25+V\sum_{i=1}^{n-1}C_{i}}{m_{0}}\times 100\%$$

Here, $C_{n}$ represents the drug concentration at time point t $\left.(\mu g\;\cdot \;{\mathrm{mL}}^{-1}\right.)$, $C_{i}$ denotes the drug concentration at the time point immediately preceding $\left.(\mu g\;\cdot \;{\mathrm{mL}}^{-1}\right.)$, $m_{0}$ is the total amount of drug in 2 mL of nanoparticles, and *V* is the sample volume (2 mL).

#### 2.4.2. Evaluation of Corneal Permeability In Vitro

The cornea’s permeability is crucial for absorbing non-invasive ocular drug formulations, becoming a key barrier to effective ocular therapy. This was assessed by utilizing the modified Franz cell diffusion method while deciphering the ex vivo corneal permeability of nanoparticles before and after CPP modification [22]. This adhered to the corneal excision techniques described in the literature [23]. The prepared rabbit corneas were positioned epithelial side up and mounted inside the diffusion cell while separating the donor and receiver compartments. The receiver compartment was filled with 5 mL of artificial aqueous humor at pH 7.4. In comparison, 0.5 mL of the test solution containing 0.1 mg of triamcinolone acetonide (TA) was applied to the donor compartment. The donor compartment was sealed using paraffin film to prevent evaporation and contamination while maintaining the setup at 34 ± 1 °C with a stirring rate of 200 rpm. Samples of 2.0 mL were obtained from the receiver at intervals of 0.5, 1, 2, 4, 6, and 8 h. Each withdrawal followed an addition of an equal volume of pre-warmed artificial medium to maintain consistent conditions. The permeation of TA was quantified using HPLC. HPLC helped determine the permeation quantity of triamcinolone acetonide by calculating the cumulative permeation quantity (*Q*), the apparent permeability coefficient ($P_{\mathrm{app}}$), and the steady-state flux ($J_{\mathrm{ss}}$). The following formula can obtain the cumulative permeation quantity:

(4) $$Q=V_{0}C_{n}+V\sum_{i=1}^{n-1}C_{i}$$

where $V_{0}$ represents the total volume of the medium in the lower chamber of the diffusion cell (5 mL), *V* denotes the volume of each sampling (2 mL), $C_{n}$ signifies the drug concentration in the lower chamber at time point t, and $C_{i}$ indicates the drug concentration in the lower chamber measured at the time point after preceding t. The given formula calculated the apparent permeability coefficient $P_{\mathrm{app}}$ ($\mathrm{cm}\;\cdot \;s^{-1})$:

(5) $$P_{app}=\frac{\Delta Q}{\left(\Delta t\times C_{0}\times A\times 60\right)}$$

$J_{ss}$, where $C_{0}$ depicts the initial drug concentration in the donor compartment, and A denotes the effective permeation area of the diffusion cell (0.5024 cm^2^). The provided formula helped calculate the steady-state flux $J_{\mathrm{ss}}\left(\mu g\;s^{-1}\;{\mathrm{cm}}^{-2}\right)$:

(6) $$J_{ss}=C_{0}P_{app}$$

### 2.5. In Vivo Study of Tear Elimination and Aqueous Humor Pharmacokinetics

#### 2.5.1. Tear Elimination

As previously stated, the tear film forms the principal barrier impeding drug penetration into the ocular surface, with tear fluid clearing most drug substances before ocular penetration. Consequently, prolonging the ocular residence time of the drug on the cornea is the most efficacious strategy to augment drug bioavailability. This study employed the filter paper technique to assess the corneal retention efficacy of nanoparticles post-CPP modification in an ex vivo rabbit model [24,25]. A cohort of 12 robust New Zealand white rabbits in the weight range of 1.5–2.5 kg and sourced from the Guangdong Provincial Medical Laboratory Animal Center was randomly assigned to four experimental groups. The Animal Ethics Committee of Guangdong Pharmaceutical University granted ethical approval for all the procedures under the reference number GDPULAC2019157. Each animal group received an equivalent dosage of nanoparticles, pre- and post-CPP modification, administered to the ocular surface. Subsequently, calibrated filter paper strips were gently introduced inside the conjunctival sac at precise time intervals of 15, 30, 60, 90, 120, and 180 min post-administration. Moreover, the eyelids were momentarily closed for 30 s to induce tear absorption. Then, the strips were promptly extracted and weighed. The collected tear mass was converted to volume using the specific gravity of 1.005 $g{\mathrm{mL}}^{-1}$. Later, 200 μL of acetonitrile was introduced inside the centrifuge tubes, vortexed for 30 s, subjected to ultrasonication for 10 min, and centrifuged at 1000 rpm for 10 min to collect the supernatant. Finally, the supernatant was analyzed using HPLC to determine the drug concentration.

#### 2.5.2. Aqueous Humor Pharmacokinetics

The aqueous humor is a vital transparent fluid in the eye, with essential functions such as maintaining the stability of intraocular pressure while providing nutrients and oxygen to the different tissues and cells of the anterior segment. It also maintains the microenvironmental balance inside the eye by clearing metabolic waste. Due to its similarities with blood, aqueous humor partially fulfills the role of blood, particularly during circulation and material exchange processes inside the eye. The circulation of aqueous humor is crucial in drug distribution and elimination within the eye. When drugs enter the eye through topical application or other routes, the aqueous humor disperses the medication to various ocular structures. The aqueous humor can carry away a drug portion, which is expelled from the body via the intraocular fluid circulation system. This reduces drug accumulation and its potential toxic effects within the eye [26]. Microdialysis sampling technology obtains aqueous humor at various time points post-administration to investigate the pharmacokinetic behavior of drugs inside the eye [27]. A total of 20% urethane was administered at 5 $\mathrm{mL}{\mathrm{kg}}^{-1}$ for rabbit anesthesia, followed by inducing mydriasis using tropicamide eye drops. A 20 G needle was inserted into the anterior chamber at the eye’s canthus, and the probe was secured after needle removal. After probe implantation within the rabbit’s eye, one probe extremity was affixed to an automated sampling apparatus. The temporal parameter was established for individual sample collection at 30 min. The opposite probe end was affixed to an injection pump, with the flow rate set to 0.5 $\mu L\;\min^{-1}$ to perfuse physiological saline for 1 h, replenishing the rabbit’s aqueous humor. The average probe recovery was determined using the reverse dialysis method [28]. The perfusion fluid was replaced using a physiological saline solution containing triamcinolone acetonide at 5 $\mu L\;\min^{-1}$ concentration. After a 1-h perfusion period to ensure system equilibration, sampling was initiated. Then, six aliquots were obtained in parallel, each with a 15 μL volume. The triamcinolone acetonide concentration in the samples was ascertained using HPLC. The formula subsequently calculated the recovery rate (R):

(7) $$R=\frac{C_{perfusion}-C_{dialysis}}{C_{perfusion}}\times 100\%$$

where $C_{perfusion}$ and $C_{dialysis}$ represent the triamcinolone acetonide concentrations in the perfusion and dialysis fluid, respectively. An in vivo pharmacokinetic study was conducted on rabbits by gently opening the eyelids and administering 180 μL of the nano-formulation. The eyelids were briefly closed to ensure ocular retention. An automated sampler helped collect samples over 6 h. All the collected samples were stored at 15 °C for subsequent analysis. The drug concentration in the aqueous humor ($C_{m}$) was determined using high-performance liquid chromatography (HPLC) based on the following formula:

(8) $$C_{m}=\frac{C_{dialysis}}{R}$$

where $C_{dialysis}$ depicts the triamcinolone acetonide concentrations in the samples, and *R* represents the probe recovery rate.

### 2.6. Evaluation of In Vitro Cytotoxicity and Transepithelial Cell Uptake

We selected MDCK cells (Beijing Beina Bioscience & Technology Co., Ltd., Beiing, China) as the model system to determine the cellular performance of nanoparticles post-CPP modification. The cells were cultured inside MEN complete medium supplemented with 1% penicillin–streptomycin (PS) and 10% fetal bovine serum (FBS). The culture flasks were placed inside an incubator to maintain a relative humidity of 95%, a carbon dioxide concentration of 5%, and a 37 °C temperature. The culture medium was refreshed every 1 to 2 days, depending on the cellular growth status, until the confluence exceeded 90%. Subsequent experiments were performed with cells from passages 10 to 20.

#### 2.6.1. Cytotoxicity

To ensure that the potential cytotoxic effects did not confound the cellular uptake behavior of Coumarin-6 dye-labeled nanoparticles (NPs), we evaluated the inhibitory effect of these NPs on the proliferation of MDCK cells with the CCK-8 assay before performing the cellular uptake experiments. Briefly, a cell suspension was prepared at approximately 5 × 10^4^ cells/mL. Subsequently, 100 μL of the cell suspension and blank culture medium were added to each of the 96-well plates. After incubating the cells under standard conditions for 24 h, the test nanoparticles labeled with Coumarin-6 were introduced into the wells. Moreover, the blank and control groups were replenished with fresh medium and further incubated for another 8 h. Then, the culture medium from the wells was aspirated. Later, 100 μL of CCK-8 solution diluted to 10% with culture medium was added to each well. This was followed by continued incubation at 37 °C for 1 to 4 h. The optical density (OD) at 450 nm of each well was measured using a microplate reader, with the OD values directly proportional to cellular viability. Therefore, the cell survival rate was measured with the following formula:

(9) $$Cell_{survivalrate}\left(\%\right)=\frac{As-Ab}{Ac-Ab}\times 100\%$$

where As represents the experimental wells containing CCK-8, the test compound, cells, and the culture medium. Ac denotes the control wells, comprising CCK-8, cells, and culture medium, without the test compound. Ab signifies the blank wells containing only CCK-8 without any additional components, where As represents the experimental wells containing CCK-8, the test compound, cells, and the culture medium. Ac denotes the control wells, comprising CCK-8, cells, and culture medium, without the test compound. Ab signifies the blank wells containing only CCK-8 without any additional components.

#### 2.6.2. Characteristics of Uptake

MDCK cells were analyzed using flow cytometry to determine the CPP modification effect on nanoparticle cellular uptake. The cells were seeded in 6-well plates and, at 90% confluence, treated with 2 μL of nanoparticle suspension using a fluorescent probe for 2 h. After removing the supernatant and washing with cold PBS, the cells were trypsinized and centrifuged at 1000 rpm. Then, the wash/centrifuge process was repeated to remove surface fluorescence. The cells were resuspended in 0.3 μL PBS for flow cytometry, and the fluorescence intensity of 1 × 10^4^ cells was measured, with uptake differences evaluated using the Mann–Whitney U test.

#### 2.6.3. Uptake Mechanism

The study investigated the cellular uptake mechanism of nanoparticles with and without CPP modification. MDCK cells were treated using different functional inhibitors before incubation with fluorescent nanoparticle probes. The cells were plated in 6-well plates and cultured until reaching 90% confluence. The functional inhibitors (listed in Table 2) were added to the MEM medium and incubated for 60 min. After removing the inhibitors and PBS washing, the cells were incubated with nanoparticles for 2 h. Finally, the medium was removed, and cells were rinsed with cold PBS, trypsinized, and analyzed using a flow cytometer.

#### 2.6.4. Preparation and Evaluation of Epithelial Cell Barrier Model

MDCK cells are characterized by a transepithelial electrical resistance (TEER) exceeding 500 $\Omega {\mathrm{cm}}^{-2}$. These are widely used to study transmembrane drug transport and their formulations [29]. Polycarbonate Transwell inserts with a pore size of 3 μm helped establish an ocular epithelial barrier model. The specific method involved harvesting MDCK cells from passages 10 to 20. A cell suspension was prepared and cultured inside an MEM complete medium for 4 to 6 days. The TEER of the Transwell inserts, without cells, served as a blank control, and the electrical resistance was measured based on the following formula:

(10) $$TEER=(R-R_{0})\times Transwellarea$$

where *R* denotes the average electrical resistance of the Transwell inserts containing cells; it signifies the average resistance of the cell-free control Transwell inserts, with a membrane surface area of 1.12 cm.

The Na-F permeability was measured using a literature-described method [30]. The MDCK cell barrier model was prepared, and the culture medium was replaced using pre-warmed PBS for rinsing. Subsequently, 1.5 milliL PBS was added to the lower chamber, and 500 μL Na-F solution was added to the upper chamber of a 12-well plate. A total of 200 μL aliquots from the lower chamber was obtained at intervals and transferred to a 96-well plate at 5, 15, 30, 60, 90, 120, and 180 min, replenishing equal volumes of PBS. Fluorescence was read using a microplate reader (Ex: 485 nm, Em: 535 nm), with cell-free Transwells as controls. The Na-F permeability ($P_{e}$) was measured using the following formula:

(11) $$P_{e}^{-1}=P_{t}^{-1}-P_{f}^{-1}$$

(12) $$P=dQ/dt\times V_{L}/A/C_{0}$$

where dQ/dt represents the flux rate of Na-F in the Transwell chamber; $V_{L}$ denotes the Transwell lower chamber volume; *A* signifies the membrane area of the Transwell upper chamber; $P_{t}$ signifies the membrane area of the Transwell upper chamber; Co refers to the initial concentration of Na-F added to the upper chamber; and $P_{f}$ depicts the permeability of the chamber without cells.

#### 2.6.5. Evaluation of Permeability Across Barriers

Before and after CPP modification, the permeation of nanoparticles through epithelial cells was assessed after the MDCK epithelial barrier model achieved the experimental criteria. In the upper chamber of the Transwell assay, 0.5 mL of the nanoparticle sample solution at 100 $\mu g{\mathrm{mL}}^{-1}$ was introduced in PBS. Simultaneously, 1.5 mL of blank PBS was added to the lower chamber and was incubated at 37 °C. Samples of 200 μL were retrieved from the receiver compartment at 15, 30, 60, 90, 120, 180, and 240 min and replaced using an equal volume of pre-warmed fresh PBS. The collected samples were mixed with 200 μL of acetonitrile, subjected to ultrasonication inside a water bath for 10 min, and centrifuged at 1000 rpm for 10 min. A 100 μL aliquot of the supernatant was obtained for analysis. The cumulative permeability quantity (*Q*) through the cellular barrier was calculated using the formula:

(13) $$Q=V_{0}C_{n}+V\sum_{i=1}^{n-1}C_{i}$$

$V_{0}$ represents the total medium volume, in the lower chamber of the Transwell (1.5 mL), *V* denotes the volume of each sample taken (0.2 mL), $C_{n}$ signifies the drug concentration in the lower chamber at time point *t*, and $C_{i}$ indicates the drug concentration in the lower chamber determined at the time point immediately preceding *t*.

#### 2.6.6. Mechanism of Crossing the Epithelial Barrier

We utilized cell function-related inhibitors and incubated nanoparticles, with and without CPP, in the Transwell epithelial cell barrier’s upper chamber to explore the CPP modification effects on nanoparticle transmembrane transport. At predefined time points, samples were obtained from the lower chamber for analysis, comparing the transport efficiency and the transmembrane mechanisms versus endocytic pathways post-cell functional characteristic alterations.

### 2.7. Data Analysis and Statistics

Initially, data were subjected to tests for normality and homogeneity of variance. When the data were normally distributed with homogeneous variances, a one-way analysis of variance (ANOVA) was conducted. In the event of statistically significant differences (*p* < 0.05), Dunnett’s method was utilized for multiple comparisons. If the data did not conform to normal distribution or variances were unequal, the Kruskal–Wallis test was used. Upon finding statistically significant differences (*p* < 0.05), the Mann–Whitney U test was utilized for pairwise comparisons.

## 3. Results and Discussion

### 3.1. Preparation and Evaluation of Triamcinolone Acetate Nanocomposite Vesicles

**Figure 1:**
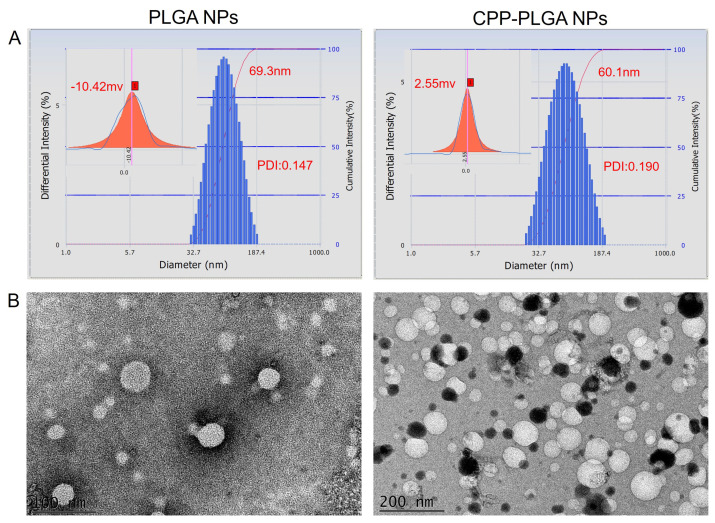
Particle size, potential, and microscopic images of NPs. (**A**) The particle size and potential of NPs. (**B**) Microscopic image of the NPs.

Ocular drug delivery systems should be non-irritating. Hence, we inspected the physical properties of the formulated eye drops. The pH was between 7.13 and 7.42, falling within the tolerable range for the eye (5.0 to 9.0). The osmotic pressure of the nanoparticles, determined using a freezing point osmometer, was between 281 and 278 mOsmol/kg before and after CPP modification, respectively. Within this range, no significant discomfort could be observed when applied ophthalmically [31]. It is essential to maintain the air–tear fluid interface’s surface tension in the physiological range of 40–46 $mNm^{-1}$ for tear film stability. Post-CPP modification, nanoparticle surface tension was elevated from 41.71 ± 0.55 to 42.06 ± 0.81 $mNm^{-1}$. This facilitated better medication spread and retention on the ocular surface, potentially extending drug retention time [32]. Fabricated PLGA nanoparticles were analyzed using a nanoparticle size/zeta potential analyzer with a PDI between 0.147 and 0.190 (Figure 1A). CPP addition refined particle size and uniformity. Transmission electron microscopy (Figure 1B) verified their spherical morphology, with diameters under 200 nm, aligning with laser size analysis and satisfying ocular application standards [33]. The incorporation of CPP was characterized by its cationic amino acid residues, which induced a significant change in the zeta potential of the nanoparticles, as shown in Table 3, shifting from (−10.9 ± 0.46) mV to (2.6 ± 0.31) mV. This reversal in surface charge improved the interaction with the negatively charged corneal mucin layer, thereby enhancing ocular permeability and nanoparticle retention [34]. Table 4 shows that the encapsulation efficiency was augmented by 7.5% after adding CPP without adversely impacting the drug loading capacity.

### 3.2. In Vitro Drug Release Study

**Figure 2:**
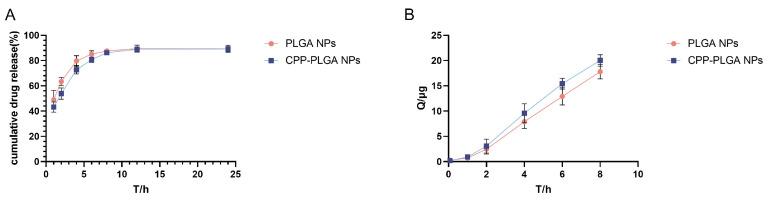
In vitro release and ex vivo corneal permeation studies. (**A**) The cumulative release rate of NPs. (**B**) In vitro corneal permeation of NPs.

The in vitro release test results in simulated aqueous humor (Figure 2A) depict that adding CPP had a negligible effect on the release behavior. The overall drug release curve showed two phases. A rapid release before 4 h was followed by a gradual stabilization as the carrier PLGA hydrated and swelled. The release behavior for the nanoparticles was fitted to four release models: zero-order, Higuchi, first-order, and Ritger–Peppas kinetics. As shown in Table 5, both types of nanoparticles accorded with the Ritger–Peppas kinetic release model.

### 3.3. Permeability Evaluation of Isolated Corneas

The ex vivo corneal permeation data, depicted in Figure 2B, reveal a monotonic increase in the cumulative drug permeation over time, showing a near-linear correlation. The CPP-PLGA NPs manifested a 13% augmentation in cumulative permeation based on culminating permeation relative to their unmodified PLGA NPs counterparts. The apparent permeability coefficient (Papp) and the steady-state flux are delineated in Table 6. This reflected a 14% escalation in Papp for the CPP-PLGA NPs compared to the unmodified formulation.

### 3.4. The Elimination of Triamcinolone Acetonide in Tears

**Figure 3:**
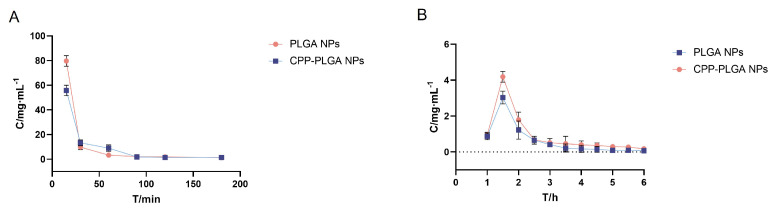
Tear elimination and aqueous humor dynamics studies. (**A**) TA elimination curve in tears. (**B**) The pharmacokinetic curves of TA within aqueous humor.

Figure 3A represents the drug concentration–time curves in tear fluid, with the $C_{max}$ and AUC listed in Table 7 for each formulation. The $C_{max}$ of PLGA NPs was 1.43 times that of the CPP-PLGA NPs, while it was 95.7% of the PLGA NPs modified with CPP. The elimination curves depict that the PLGA NP concentration was significantly higher than CPP-PLGA NPs at 15 min (*p* < 0.01). The concentration rapidly declined to a level lower than the CPP-PLGA NPs after 15 min (*p* < 0.05) until there was no statistically significant difference between the two at 90 min. Thus, nanoparticles modified with CPP depict better adhesion to ocular tissues post-administration while being eliminated by tear fluid at a slower rate than unmodified nanoparticles. CPP is a positively charged short peptide, which adsorbs onto the surface of PLGA NPs through electrostatic attraction [35] to produce CPP-PLGA NPs. The $C_{max}$ of CPP-PLGA NPs was reduced compared to PLGA NPs. At the same time, the AUC was slightly higher, which could be due to the CPP addition rendering the nanoparticles positively charged and adhering to the ocular surface. CPP-PLGA NP retention on the ocular surface resulted from improved surface adhesion and intensified tear fluid elimination.

### 3.5. Pharmacokinetic Study of In Vivo Aqueous Humor

The intraocular drug concentration–time curves are shown in Figure 3B, where formulations achieved their peak concentrations at 1.5 h. Ocular pharmacokinetic studies include non-compartmental and compartmental models. This study employed a non-compartmental model to determine the pharmacokinetic parameters. Pharmacokinetic data were processed using pharmacokinetic software (Table 8). The $C_{max}$ of CPP-PLGA NPs was 4.19 $\mu g{\mathrm{mL}}^{-1}$, which was a 38% increase compared to the NPs without CPP modification (3.03 $\mu g{\mathrm{mL}}^{-1}$). The $AUC_{0\to 360}$ (5.05 μg/mL·min) depicted a 46% increase (3.46 μg/mL·min), suggesting that the modification with CPP improved the nanoparticles’ corneal permeability. After 2 h, the intraocular drug concentration in the CPP-PLGA NPs group noticeably declined more gradually than in the PLGA NPs group. The $C_{max}$ and $AUC_{0\to 360}$ of the CPP-PLGA NPs were higher than those of the PLGA NPs. This indicated better drug absorption, higher bioavailability, and superior drug release characteristics.

### 3.6. Mechanisms of Cellular Uptake

**Figure 4:**
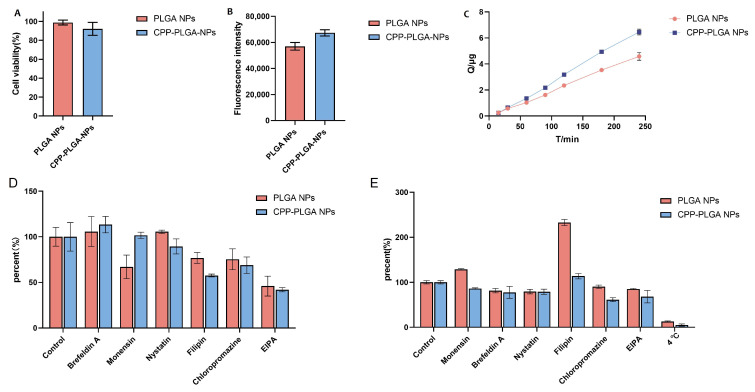
Properties and mechanisms of NPs transport across cellular barriers The data are given as mean ± SD. (**A**) The cytotoxicity study of coumarin-6 nanoparticles. (**B**) The difference in NP uptake by MDCK cells. (**C**) Epithelial barrier penetration studies of NPs. (**D**) The mechanism analysis of endocytosis of MDCK cells monolayer for nanoparticles with different properties by adding various inhibitors. (**E**) The mechanism analysis of NP transport across the epithelial cell barrier by adding different inhibitors.

The cytotoxicity results are shown in Figure 4A, which indicate that the cell viability before and after CPP modification for nanoparticles loaded with coumarin-6 remained above 90%. These data indicate that the prepared fluorescent probes exhibited low cytotoxicity. Further observation of the MDCK cell uptake of nanoparticles is depicted in Figure 4B, where CPP-modified nanoparticles showed a significant elevation in cellular uptake compared to nanoparticles without such modification (*p* < 0.01). The uptake characteristics of the NPs are illustrated in Figure 4D. The experimental results depict that the PLGA NP uptake primarily occurred through macropinocytosis, with Golgi apparatus-mediated endocytosis participating in the uptake process. The Golgi apparatus also played a role in nanoparticle expulsion. For the CPP-PLGA NPs, the results confirm that they were primarily internalized through macropinocytosis, with caveolin/lipid raft-mediated endocytosis and clathrin-mediated endocytosis playing auxiliary roles. Notably, the Golgi apparatus inhibition did not significantly affect the fluorescence intensity of the cells. Therefore, CPP modification enables the nanoparticles to bypass the Golgi apparatus function during intracellular transport. Compared to the unmodified PLGA NPs, CPP modification significantly improved caveolin/lipid raft-mediated endocytosis but decreased the role of the Golgi apparatus (*p* < 0.05). The permeability of Na-F reached ($6.27\pm 1.48)\times 10^{-6}\;\mathrm{cm}\min^{-1}$. The MDCK epithelial barrier constructed showed a high transepithelial electrical resistance (TEER) value and a low Na-F permeability rate, depicting a high tightness between MDCK cells and successful barrier formation. Based on the epithelial cell barrier permeation experimental results in Figure 4C, the average permeation of the CPP-PLGA NPs within 4 h was 41% higher than unmodified PLGA NPs. This finding is consistent with the trend of the cellular uptake experiment, suggesting a positive correlation between nanoparticle uptake and their permeation via the cellular barrier. Thus, CPP modification significantly improved the ability of nanoparticles to penetrate the epithelial cell barrier, further validating the CPP potential in enhancing the efficiency of drug delivery systems. The results of the transmembrane transport mechanism are represented in Figure 4E. The study results depict that the transmembrane transport of nanoparticles with different characteristics was significantly inhibited under low-temperature conditions (4 °C), demonstrating energy dependence. For the PLGA NPs, the effects of different drugs induced changes in transmembrane transport quantity opposite to their uptake trends. Brefeldin A may be related to the Golgi apparatus, affecting endocytosis and exocytosis processes. In contrast, nystatin may affect transmembrane transport via caveolin/lipid raft-mediated endocytosis. Chlorpromazine and EIPA suppressed transmembrane transport, indicating the involvement of endocytosis and macropinocytosis. Filipin enhanced the transmembrane transport quantity, which may be associated with the improved uniformity of cholesterol distribution results. For the CPP-PLGA NPs, Monensin and Brefeldin A showed inhibitory effects on transmembrane transport, which were closely associated with the Golgi apparatus. Nystatin, Chlorpromazine, and EIPA also showed inhibitory effects on transmembrane transport, consistent with the uptake trend, suggesting the role of endocytosis and macropinocytosis. The impact on Filipin was small, possibly due to the CPP modification improving cholesterol distribution uniformity. Integrating the above results, endocytosis and macropinocytosis play a role in PLGA nanoparticles. In contrast, CPP modification enhances the role of transmembrane transport pathways while affecting cholesterol distribution uniformity. There is a certain relationship between the transmembrane transport of nanoparticles and cellular uptake, but there is no correlation.

## 4. Conclusions

This study synthesized and characterized CPP-modified PLGA nanoparticles for ocular drug delivery. The formulation revealed minimal irritation and improved delivery of the model drug TA to the eye. CPP modification enhanced the nanoparticles’ retention and corneal penetration, extending the drug’s duration within the aqueous humor. Furthermore, coumarin-6-loaded nanoparticles indicated that CPP facilitates cellular uptake and trans-barrier transport. The current research validates the potential of CPP-modified nanoparticles in enhancing ocular drug delivery and retention. The study offers insights for developing effective drug delivery systems and overcoming biological barriers.

## Figures and Tables

**Table 1:** The composition of nanoparticle eye drops with different characteristics (%).

Components	PLGA NPs	CPP-PLGA NPs
TA/coumarin-6	0.02	0.02
PLGA	0.2	0.2
PVA	2	2
2-HP-β-CD	1.5	1.5
CPP	-	0.04
deionized water	97.28	96.24

TA: triamcinolone acetonide; PLGA: Poly(lactic-co-glycolic acid); PVA: polyvinyl alcohol; 2-HP-β-CD: 2-Hydroxypropyl-β-cyclodextrin; CPP: Cell-penetrating peptide.

**Table 2:** The concentrations of different inhibitors for mechanism research.

Inhibitor/Enhancer	Concentration
Filipin	0.125 $\mu g{\mathrm{mL}}^{-1}$
Nystatin	30 $\mu \mathrm{mol}L^{-1}$
EIPA	40 $\mu \mathrm{mol}L^{-1}$
Brefeldin A	25 $\mu g{\mathrm{mL}}^{-1}$
Monensin	32.5 $\mu g{\mathrm{mL}}^{-1}$
Chloropromazine	30 $\mu \mathrm{mol}L^{-1}$

**Table 3:** The zeta potential measurement (mean ± SD, *n* = 3).

Sample	Zeta Potential (mV)
PLGA NPs	−10.9±0.46
CPP-PLGA NPs	2.6±0.31

**Table 4:** The encapsulation efficiency (EE) and drug load (DL) measurement (mean ± SD, *n* = 3).

Sample	EE (%)	DL (%)
PLGA NPs	73.73±4.8	7.39±0.48
CPP-PLGA NPs	79.25±5.5	7.56±0.54

EE: encapsulation efficiency; DL: drug loading.

**Table 5:** Fitting results of in vitro release model of nanoparticles with different surface characteristics.

NPs	Release Model	Fitting Equation	$R^{2}$
PLGA NPs	Ritger–Peppas	ln(Q)=0.1939×ln(t)+4.0101	0.8303
CPP-PLGA NPs	Ritger–Peppas	ln(Q)=0.2471×ln(t)+3.8567	0.8827

**Table 6:** $P_{app}$ and $J_{ss}$ of nanoparticles in excised corneal penetration test (mean ± SD, *n* = 3).

Parameter	$P_{\mathrm{app}}\times 10^{6}$ (cm s^−1^)	$J_{\mathrm{ss}}\times 10^{3}\;\left(\mu {g\;s}^{-1}\right)$ cm^−2^
PLGA NPs	6.63±0.27	1.33±0.05
CPP-PLGA NPs	7.52±0.35	1.52±0.07

$P_{app}$: apparent permeability coefficient; $J_{ss}$: steady-state flux.

**Table 7:** The parameters related to triamcinolone elimination in tears (mean ± SD, *n* = 6).

Parameter	PLGA NPs	CPP-PLGA NPs
$C_{max}$ (μg/mL)	79.65±4.39	55.71±4.23
$AUC_{0\to 180}\;\left(\mu g/mL\;\cdot \;\min\right)$	1100.43±122.75	1148.61±174.43

$C_{max}$: maximum concentration; AUC: area under the curve.

**Table 8:** The pharmacokinetic parameters of triamcinolone in aqueous humor (mean ± SD, *n* = 3).

NPs	$C_{\max}\;\left(\mu {g\;\mathrm{mL}}^{-1}\right)$	${\mathrm{AUC}}_{0\to 360}\;\left(\mu g/\mathrm{mL}\;\cdot \;\min\right)$	MRT (h)
PLGA NPs	3.03±0.35	3.46±0.20	2.02±0.12
CPP-PLGA NPs	4.19±0.31	5.05±0.31	2.24±0.13

MRT: mean residence time.

## Data Availability

The datasets used or analyzed during the current study are available from the corresponding author upon reasonable request.
